# Primary Biliary Cholangitis‐Associated Granulomatous Interstitial Lung Disease: Pulmonary Epithelial‐Interface Injury Supporting the Concept of Generalized Autoimmune Epithelitis

**DOI:** 10.1111/pin.70166

**Published:** 2026-08-19

**Authors:** Jiayao Liu, Yuki Kamikokura, Manami Hayashi, Akira Sakata, Naoko Aoki, Miki Saito, Yoshinori Minami, Takaaki Sasaki, Masahiro Kitada, Yukio Nakatani, Sayaka Yuzawa, Shin Ichihara, Mishie Tanino

**Affiliations:** ^1^ Department of Diagnostic Pathology Asahikawa Medical University Hospital Asahikawa Japan; ^2^ Department of Radiology Asahikawa Medical University Asahikawa Japan; ^3^ Division of Cardiology, Nephrology, Pulmonology and Neurology, Department of Internal Medicine Asahikawa Medical University Asahikawa Japan; ^4^ Division of Vascular, Respiratory and Surgical Oncology, Department of Surgery Asahikawa Medical University Asahikawa Japan; ^5^ Department of Pathology Yokosuka Kyosai Hospital Yokosuka Japan

**Keywords:** autoimmune epithelitis, epithelial‐interface injury, interstitial lung disease, non‐caseating granulomas, primary biliary cholangitis

## Abstract

Primary biliary cholangitis (PBC) can be accompanied by interstitial lung disease (ILD) manifesting as granulomatous interstitial lung disease, but the mechanism of granuloma formation remains unclear. We report a case of PBC‐ILD coexisting with anti‐synthetase syndrome (ASS) in a 49‐year‐old woman who presented with persistent cough, facial rash, and arthritis. Laboratory tests demonstrated cholestatic enzyme elevation, anti‐mitochondrial M2 positivity, and anti‐aminoacyl‐tRNA synthetase antibody positivity, with elevated KL‐6 and SP‐D levels. High‐resolution CT revealed lower‐lobe‐predominant ground‐glass opacities. Bronchoalveolar lavage fluid showed lymphocytosis with high CD4/CD8 ratio. Liver biopsy confirmed PBC. Surgical lung biopsies revealed epithelial‐interface injury and focal apoptosis of bronchiolar and alveolar epithelium accompanied by CD4‐dominant T‐cell infiltration and non‐caseating granulomas (NCGs). Segment 2 showed cellular NSIP pattern and segment 9 showed fibrotic NSIP pattern. NCGs were more prominent in the cellular NSIP areas and decreased in the fibrotic NSIP areas. These findings suggest a possible pathological continuum from active inflammation by epithelial‐interface injury to fibrotic remodeling. These observations may highlight the potential relevance of generalized autoimmune epithelitis in the pathogenesis of PBC‐ILD.

## Graphical abstract

We report a rare case of primary biliary cholangitis‐associated granulomatous interstitial lung disease (PBC‐GILD) in a patient with coexisting anti‐synthetase syndrome. Bronchoalveolar lavage fluid revealed lymphocytosis with multinucleated giant cells. Lung biopsy showed nonspecific interstitial pneumonia (NSIP) ranging from cellular to fibrotic pattern, with non‐caseating granulomas. Immunohistochemical staining revealed cleaved caspase‐3 (CC3)‐positive cells corresponding to AE1/AE3‐positive bronchiolar and alveolar epithelium. Pulmonary epithelial‐interface injury supports the concept of generalized autoimmune epithelitis in PBC.

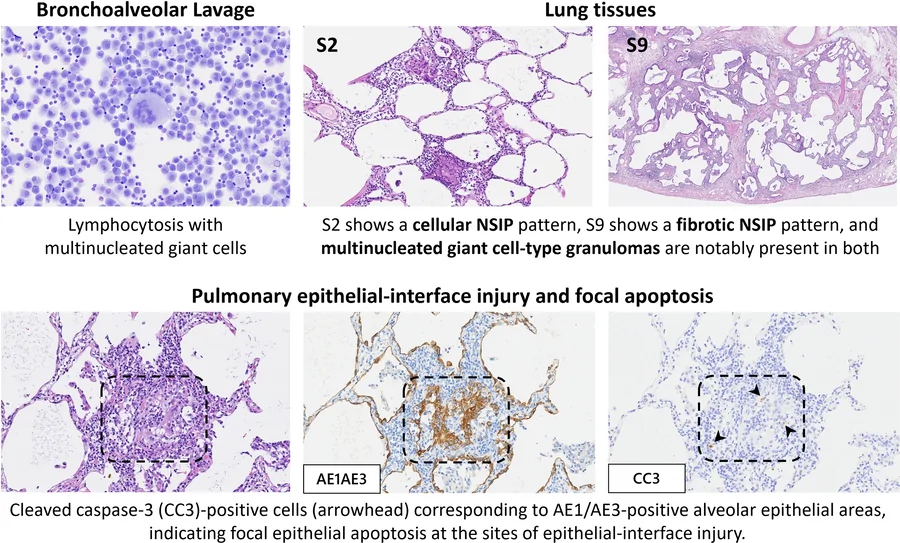

**Abbreviations**
ALP: Alkaline phosphatase
AMA: Antimitochondrial antibody
ARS: Aminoacyl‐tRNA synthetase
ASS: Anti‐aminoacyl‐tRNA synthetase syndrome
BALF: Bronchoalveolar lavage fluid
BEC: Biliary epithelial cell
CNSDC: Chronic non‐suppurative destructive cholangitis
fHP: Fibrotic hypersensitivity pneumonitis
GILD: Granulomatous interstitial lung disease
HP: Hypersensitivity pneumonitis
HRCT: High‐resolution computed tomography
ILD: Interstitial lung disease
KL‐6: Krebs von den Lungen‐6
LIP: Lymphoid interstitial pneumonia
NCGs: Non‐caseating granulomas
NSIP: Nonspecific interstitial pneumonia
OP: Organizing pneumonia
PBC: Primary biliary cholangitis
PDC‐E2: The E2 subunit of the pyruvate dehydrogenase complex
SP‐D: Surfactant protein D
UIP: Usual interstitial pneumonia
VATS: Video‐assisted thoracoscopic surgery
γ‐GTP: γ‐glutamyl transpeptidase

## 1 Introduction

Primary biliary cholangitis (PBC) is a chronic autoimmune liver disease that predominantly affects middle‐aged women [1]. It is characterized by chronic non‐suppurative destructive cholangitis (CNSDC), often accompanied by non‐caseating epithelioid granulomas within the liver. Beyond hepatic involvement, PBC is frequently associated with systemic extrahepatic manifestations, including rheumatologic, endocrine, gastrointestinal, dermatologic, and pulmonary diseases [2]. The most common pulmonary histological pattern is granulomatous interstitial lung disease (GILD) [3, 4, 5] which may mimic other GILDs, such as hypersensitivity pneumonitis and sarcoidosis.

Anti‐synthetase syndrome (ASS) is characterized by the presence of anti‐aminoacyl‐tRNA synthetase (anti‐ARS) antibodies and clinical manifestations such as rash, myositis, arthritis, and ILD. Although ASS‐ILD is a well‐established manifestation, its histopathological patterns are typically nonspecific interstitial pneumonia (NSIP), organizing pneumonia (OP), or a combination of NSIP and OP but not GILD in ASS [6]. We report a rare case of coexistence of PBC and ASS and explore the mechanisms and pathogenesis of ILD.

## 2 Clinical Summary

**Figure 1:**
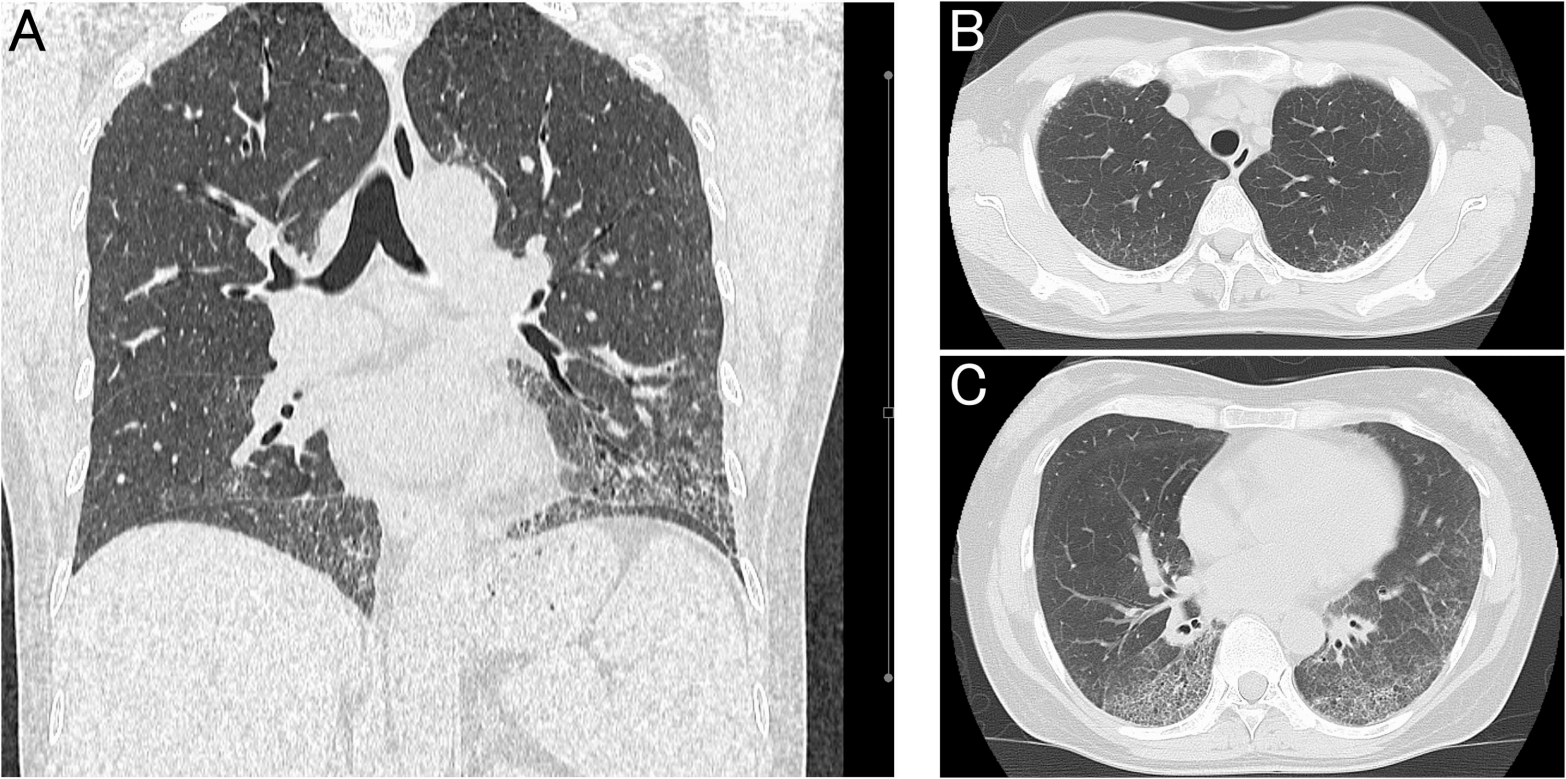
HRCT of the chest. (A) Coronal view shows bilateral peripheral ground‐glass opacities with granular structures, traction bronchiectasis, and reduced lung volume, predominantly in the lower lobes. (B) Axial view of the upper lobe shows subtle subpleural granular opacities. (C) Axial view of the lower lobe demonstrates marked volume loss with reticular opacities and ground‐glass attenuation, consistent with a fibrotic NSIP pattern.

A 49‐year‐old woman presented with a persistent cough without dyspnea and no occupational or environmental antigen exposure. Pulmonary function testing demonstrated a restrictive ventilatory defect. She also presented with a facial rash and arthritis. Raynaud's phenomenon, mechanic's hands, and myositis were not observed. Laboratory examination showed cholestatic enzyme elevation, including alkaline phosphatase (ALP: 1968 U/L) and γ‐glutamyl transpeptidase (γ‐GTP: 505 U/L), with positive antimitochondrial antibody (AMA) and its M2 subtype (AMA‐M2). Krebs von den Lungen‐6 (KL‐6, 1242 U/mL) and surfactant protein D (SP‐D, 293.4 ng/mL) were elevated. Autoimmune serology was positive for antinuclear antibody and anti‐ARS antibody, with negative results for anti‐Jo‐1 and anti‐MDA5 antibodies. High‐resolution computed tomography (HRCT) revealed lower lobe‐predominant ground‐glass opacities, reticulation, traction bronchiectasis, and volume loss, with fine granular and micronodular opacities in the upper lobes along vascular bundles and lymphatic routes (Figure 1). These findings were compatible with fibrotic NSIP pattern. Bronchoalveolar lavage fluid (BALF) showed lymphocytosis (27%) with a markedly elevated CD4/CD8 ratio (8.98), and occasional multinucleated giant cells were also noted.

Immunosuppressive therapy and ursodeoxycholic acid were initiated 1 year after presentation. Over the 10‐year follow‐up period, the patient has remained stable without exacerbations of ILD.

## 3 Pathological Findings

A liver biopsy was performed, followed 3 days later by surgical lung biopsies (SLB). Histopathological examination of the liver biopsy showed CNSDC and non‐caseating epithelioid granulomas, confirming the diagnosis of PBC.

**Figure 2:**
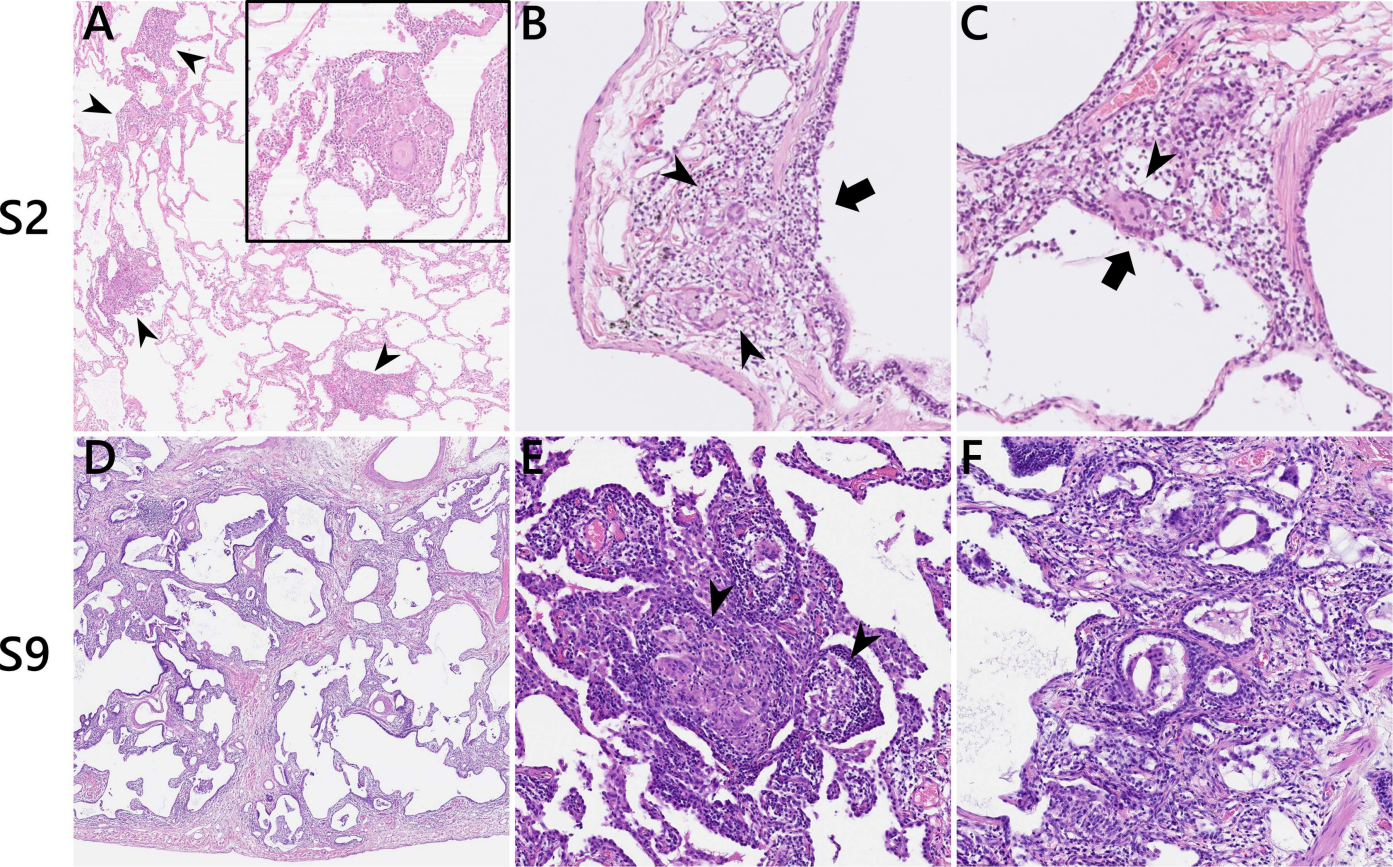
Histopathological findings from the right upper lobe (S2) and lower lobe (S9) obtained via VATS. (A–C) H&E staining of the S2 segment. (A) Cellular NSIP pattern characterized by diffuse interstitial lymphocytic infiltration without fibrosis and multiple poorly formed NCGs (arrowhead) (original magnification ×5). The magnified area in the upper right corner shows multinucleated giant cell‐like morphology within the NCGs (original magnification ×20). (B–C) Epithelial‐interface injury (original magnification ×10). (B) Granulomatous inflammation involving and disrupting the bronchiolar epithelium (arrow) (C) Granulomatous inflammation involving and disrupting the alveolar epithelium (arrow). (D–F) H&E staining of the S9 segment. (D) Histologic progression from cellular to fibrotic NSIP pattern, with increased collagen deposition replacing lymphocytic interstitial inflammation (original magnification ×5). (E) Poorly formed multinucleated giant‐cell granulomas (arrow head) similar to those observed in S9 (original magnification ×20). (F) Multinucleated giant cells containing cholesterol clefts within the bronchiolar and alveolar spaces (original magnification ×10).

Lung biopsies were obtained from segment 2 (S2) and segment 9 (S9) of the right lung (Figure 2). S2 showed uniform lymphoplasmacytic infiltration throughout the alveolar septa, without evidence of fibrosis, consistent with a cellular NSIP pattern. In some regions, epithelial injury at the bronchioles and alveoli was observed, accompanied by poorly formed NCGs containing multinucleated giant cells. NCGs were frequently distributed in the interstitium, predominantly around distal bronchioles and alveolar septa, and were also occasionally observed in subpleural areas.

**Figure 3:**
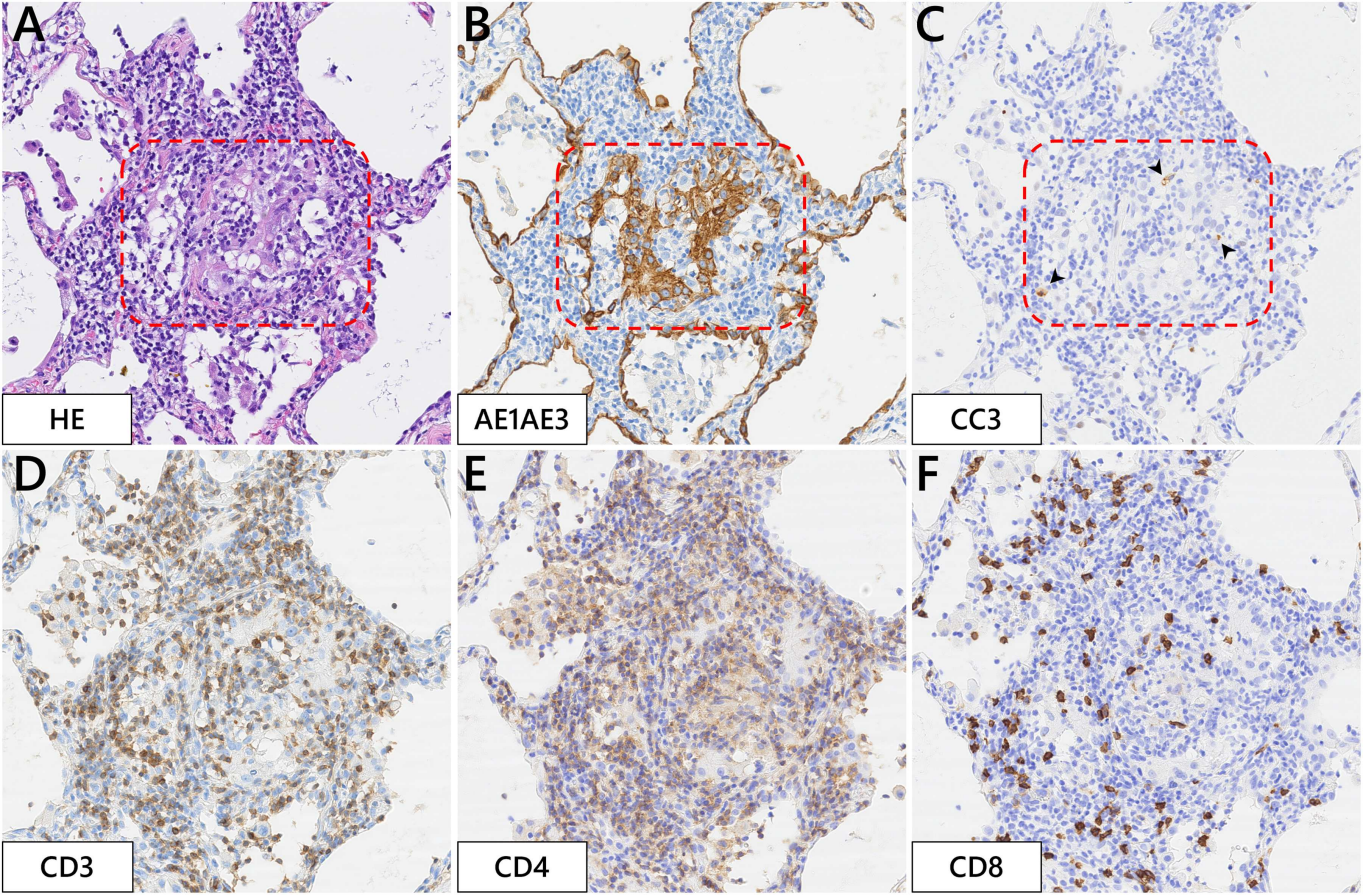
Serial‐section comparison of epithelial‐interface injury and focal epithelial apoptosis in the S2 lesion. (20x magnification). (A) H&E staining showing an inflammatory infiltrate associated with disruption of the alveolar epithelium, presenting as epithelial‐interface injury. (B) AE1/AE3 immunostaining on a serial section highlighting the corresponding alveolar epithelial lining. (C) Cleaved caspase‐3 (CC3) immunostaining on a serial section showing scattered apoptotic cells corresponding to the AE1/AE3‐positive alveolar epithelial area; (D–F) Immunohistochemical staining of epithelial‐interface injury area. (D) Diffuse CD3‐positive cells. (E) Predominant CD4‐positive cells. (F) Sparse CD8‐positive cells.

Immunohistochemical analysis showed scattered cleaved caspase‐3 (CC3)‐positive cells within AE1/AE3‐positive bronchial and alveolar epithelium, suggesting focal apoptosis of epithelial cells (Figure 3). The inflammatory infiltrates at the sites of epithelial injury at the bronchioloalveolar interface were predominantly composed of CD3‐positive T cells, with CD4‐positive cells outnumbering CD8‐positive cells. Digital image analysis showed a high CD4/CD8 ratio (range, 5.68–9.70) in these inflammatory areas (Supplementary Tables 1–3; Supplementary Figure 1), which is consistent with BALF findings. CD20‐positive B cells and CD138‐positive plasma cells were only sparsely detected.

S9 showed a predominantly fibrotic NSIP pattern, with less inflammatory infiltration and fewer poorly formed NCGs containing multinucleated giant cells compared to the findings in S2. Bronchiolitis with epithelial injury at the bronchiolar and alveolar interface was only focally observed. Multinucleated giant cells containing cholesterol clefts were observed within the alveolar spaces. No apparent vascular lesions, such as granulomatous vasculitis or vascular remodeling, were identified in either specimen.

## 4 Discussion

PBC can be accompanied by ILD, with a reported prevalence of 15.7–40.9% [3, 4]. However, histopathological characterization of PBC‐ILD remains limited because these reports rely on clinical and radiological assessments rather than lung tissue evaluation. We reviewed 47 published cases of PBC‐ILD with lung histopathology sufficiently described to permit pattern assignment to contextualize our findings [3, 4, 5, 7, 8, 9, 10, 11, 12, 13, 14, 15, 16, 17], (Table 1). GILD was the most frequently reported pattern in PBC‐ILD, followed by NSIP, LIP, OP, and UIP, often in combination. Within this spectrum, PBC‐ILD cases reported by Cavazza et al. [11] and Lee et al. [5] closely resemble our case, featuring multinucleated giant cell‐rich NCGs on an NSIP background.

**Table 1:** Summary of cases of PBC‐ILD with documented lung biopsy histopathology.

Year	Author	Total cases	Procedures	Non‐caseating granuloma	Interstitial pneumonia	Combined autoimmune disease
**1983**	Weissman et al. [15]	1	TBLB	Present	LIP	—
**1987**	Wallace et al. [14]	4	LB	Present (*n* = 1)	Lymphocytic bronchiolitis (*n* = 1)	SS (*n* = 4)
**1998**	Strobel et al. [13]	1	LB	Present	LIP + OP	—
**2008**	Ishii et al. [12]	1	LB	Absent	UIP+ honeycomb	—
**2008**	Liu et al. [8]	5	LB	Absent	NSIP (*n* = 5)	SS (*n* = 4)
**2009**	Shen et al. [3]	5	LB	Absent	LIP (*n* = 3), NSIP (*n* = 2)	
**2010**	Cavazza et al. [11]	1	TBLB	Present	NSIP	—
**2012**	Bartosiewicz et al. [10]	2	SLB	Present (*n* = 1)	LIP (*n* = 1)	—
**2013**	Asato et al. [16]	1	SLB	Absent	UIP	
**2015**	Laudenbach et al. [9]	1	TBLB, SLB	Present	LIP	—
**2015**	Franco et al. [7]	1	TBLC	Present	NSIP	—
**2018**	Lee et al. [5]	16	SLB, TBLB, lobectomy, CNB	Present (*n* = 13)	OP (*n* = 5), NSIP (*n* = 4), UIP (*n* = 2)	—
**2023**	Kalashnikov et al. [4]	7	SLB	Present (*n* = 7)	OP (*n* = 2)	—
**2024**	Feng et al. [17]	1	LB	Present	NSIP	—
**2025**	Our case	1	VATS	Present	NSIP	ASS

Abbreviations: ASS, Anti‐synthetase syndrome; BOOP, Bronchiolitis obliterans organizing pneumonia (now commonly referred to as organizing pneumonia); CNB, Core needle biopsy; LB, Lung biopsy; LIP, Lymphoid interstitial pneumonia; NSIP, Nonspecific interstitial pneumonia; OP, Organizing pneumonia. SLB, Surgical lung biopsy; SS, Sjögren's syndrome; TBLB, Transbronchial lung biopsy; TBLC, Transbronchial lung cryobiopsy; UIP, Usual interstitial pneumonia; VATS, Video‐assisted thoracoscopic surgery biopsy.

Although ILD is a well‐established manifestation of coexisting ASS, the findings in our case were not fully consistent with typical ASS‐ILD. The markedly elevated BAL CD4/CD8 ratio was inconsistent with the characteristically low ratio reported in ASS‐ILD and granulomatous inflammation is not a typical feature of ASS‐ILD [6]. Careful differential diagnosis of other GILDs is also essential. Fibrotic hypersensitivity pneumonitis (fHP) was an important differential consideration given overlapping histological features [18]. However, HRCT findings were indeterminate for fHP according to international guidelines, and no convincing antigen exposure was identified. Sarcoidosis was also considered but the granulomas differed in both distribution and morphology. Infection was excluded based on negative cultures of bacteria and drug‐related causes were excluded as no suspected drug was identified.

In PBC, bile duct injury is characterized as a targeted autoimmune epithelitis, in which biliary epithelial cells (BECs) actively participate in sustaining immune‐mediated injury [19]. A pivotal mechanism involves the failure of glutathionylation during BEC apoptosis, which allows epitopes of the E2 subunit of the pyruvate dehydrogenase complex (PDC‐E2) to remain immunologically intact within apoptotic bodies, also referred to as apotopes, thereby linking epithelial cell death to the perpetuation of autoimmune responses [19]. In the liver, this process manifests as CNSDC. Hepatic granulomas in PBC are topographically associated with damaged bile ducts and decrease in number as bile duct loss progresses, suggesting that granuloma formation represents a secondary organized immune response to epithelial injury rather than the primary injurious event [20]. This concept has been further extended beyond the liver, giving rise to the notion of PBC as a generalized autoimmune epithelitis [19].

In the present case, we offer a pathology‐based opportunity to explore this possibility in the lung, particularly as a possible form of pulmonary epithelial interface injury. S2 more clearly demonstrated active epithelial injury at the bronchiolar and alveolar interface, where AE1/AE3 and CC3 staining showed focal epithelial apoptosis, accompanied by CD4‐rich T‐cell infiltration. This finding was associated with a cellular NSIP pattern and numerous poorly formed NCGs distributed in the alveolar septa and distal bronchiolar interstitium. The close spatial association among epithelial interface injury, inflammatory infiltration, and NCGs supports the interpretation that granuloma formation may represent an organized immune response to epithelial injury, rather than the primary injurious event, consistent with the liver findings in PBC.

S9 also showed epithelial injury at the bronchiolar and alveolar interface and poorly formed NCGs. However, inflammatory infiltration and NCGs were less prominent than in S2, and the lesion showed a predominantly fibrotic NSIP pattern with cellular‐to‐fibrotic transitional areas. Multinucleated giant cells containing cholesterol clefts within the alveolar spaces may represent a secondary reaction related to prior epithelial‐interface injury [20]. Taken together, the findings in S2 and S9 may represent sequential phases ranging from active epithelial‐interface injury to fibrotic remodeling.

In conclusion, this case provides a pathology‐based perspective on PBC‐ILD, suggesting that granuloma formation may reflect epithelial‐interface injury in the lung. We therefore propose epithelial‐interface injury as a common histologic denominator of generalized autoimmune epithelitis across organs in PBC.

## Author Contributions

J.L. summarized the clinical records, contributed to the pathological diagnosis, prepared and drafted the manuscript. M.T. supervised the histopathological interpretation, confirmed the final diagnosis, and guided manuscript writing. S.I., S.Y., Y.K., M.H., A.S., and N.A. provided pathological feedback. M.S. contributed radiological analysis and feedback. Y.M. approved the use of clinical information and obtained informed consent from the patient. Y.M. and T.S. provided clinical feedback; M.K. performed VATS. Y.N. provided critical insight and proposed mechanistic hypotheses. All authors have reviewed and approved the final version of the manuscript.

## Ethics Statement

This study is a retrospective case report and does not involve any prospective research procedures requiring ethics committee approval. This case report has consent from patient to publication.

## Conflicts of Interest

Prof. Tanino Mishie is an Editorial Board Member of Pathology International and a corresponding author of this article. To minimize bias, Prof. Tanino Mishie was excluded from all editorial decision‐making related to the acceptance of this article for publication.

## Supporting information


Supporting File

## Data Availability

The data that support the findings of this study are available on request from the corresponding author. The data are not publicly available due to privacy or ethical restrictions.
