# Supplementary material for: Primary Biliary Cholangitis‐Associated Granulomatous Interstitial Lung Disease: Pulmonary Epithelial‐Interface Injury Supporting the Concept of Generalized Autoimmune Epithelitis
Source: Pathol Int. 2026 Aug 19;76(8):e70166. doi: 10.1111/pin.70166 (PMC13490269; doi:10.1111/pin.70166)
Supplement: Supplementary file 1 [file PIN-76-0-s001.docx]

Representative HALO™ analysis images showing positive membrane detection for CD4 (A) and CD8 (B) in serial sections of the lung VATS specimen. The analyzed region of interest corresponded to an epithelial-interface injury-associated inflammatory area with poorly formed granulomatous infiltration. Positive cells are highlighted by the HALO™. Scale bars: 50 μm.

**Supplementary Figure 1** HALO™-based quantitative analysis of CD4⁺ and CD8⁺ cells.

**Supplementary Table 1** HALO™ output parameters for CD4⁺ and CD8⁺ cell quantification

| **Marker** | **Tissue area (mm²)** | **Total cells** | **Positive cells** | **Positive cells / total cells** | **Positive cell density** |
| --- | --- | --- | --- | --- | --- |
| CD4 | 0.14054 | 1105 | 357 | 32.31% | 2540.20 cells/mm² |
| CD8 | 0.12584 | 886 | 56 | 6.32% | 444.85 cells/mm² |

**Supplementary Table 2** Calculation of the CD4/CD8 ratio

| **Calculation method** | **CD4/CD8 ratio** |
| --- | --- |
| Raw positive membrane count | 357 / 56 = **6.38** |
| % positive membranes | 32.31 / 6.32 = **5.11** |
| Positive cell density | 2540.20 / 444.85 = **5.71** |

**Supplementary Table 3** CD4/CD8 ratio by epithelial interface injury areas

| Epithelial interface injury area | Marker | Tissue area (mm²) | Total cells | Positive cells | Positive cells / total cells | Positive cell density | CD4/CD8 ratio |
| --- | --- | --- | --- | --- | --- | --- | --- |
| 1 | CD4 | 0.14054 | 1105 | 357 | 32.31% | 2540.20 cells/mm² | 5.71 |
|  | CD8 | 0.12584 | 886 | 56 | 6.32% | 444.85 cells/mm² |  |
| 2 | CD4 | 0.20522 | 1629 | 949 | 58.26% | 4624.31 cells/mm² | 9.70 |
|  | CD8 | 0.21594 | 1314 | 103 | 7.84% | 476.98 cells/mm² |  |
| 3 | CD4 | 0.05497 | 382 | 180 | 47.12% | 3274.51 cells/mm² | 5.68 |
|  | CD8 | 0.05032 | 312 | 29 | 9.29% | 576.31 cells/mm² |  |
| 4 | CD4 | 0.12520 | 1166 | 484 | 41.51% | 3865.81 cells/mm² | 6.17 |
|  | CD8 | 0.13567 | 1168 | 85 | 7.28% | 626.52 cells/mm² |  |
